# CLAG-M/I方案治疗儿童复发/难治急性髓系白血病的临床疗效和安全性分析

**DOI:** 10.3760/cma.j.issn.0253-2727.2022.04.013

**Published:** 2022-04

**Authors:** 慧敏 李, 娅萍 王, 婕 黄, 瑶 薛, 晓燕 孙, 拥军 方

**Affiliations:** 南京医科大学附属儿童医院血液肿瘤科，南京 210003 Department of Hematology and Oncology, Children's Hospital of Nanjing Medical University, Nanjing Medical University, Nanjing 210003, China

儿童复发/难治急性髓系白血病（AML）往往对一线治疗药物耐药，最终预后极差。因此，寻找更为有效安全的化疗方案再次获得缓解并桥接移植，对于改善这部分儿童的预后至关重要。2002年，波兰成人白血病协作组（PALG）首次使用CLAG-M（克拉屈滨、大剂量阿糖胞苷、米托蒽醌及G-CSF）方案治疗成人复发/难治AML患者，取得了较高的缓解率[1]。我国复发/难治AML指南亦推荐对一般情况及耐受性好的患者采用包括CLAG±M/I（克拉屈滨、阿糖胞苷、G-CSF，可加用米托蒽醌或去甲氧柔红霉素）方案在内的强烈化疗方案[2]。然而，目前儿童复发/难治AML尚缺乏统一的化疗方案。因此我们基于以上的临床研究及指南，采用CLAG-M/I方案治疗儿童复发/难治AML，观察这部分患者的临床特征及治疗反应，以评估该方案在儿童患者中的疗效和安全性。

## 病例与方法

1. 病例：2019年2月至2021年7月南京医科大学附属儿童医院9例确诊为复发/难治AML的儿童患者纳入研究。AML的诊断标准依据2016年WHO髓系肿瘤和急性白血病分类修订版[3]，根据骨髓细胞形态学、免疫学、细胞遗传学、分子生物学（MICM）确诊。复发性AML诊断标准：完全缓解（CR）后外周血再次出现白血病细胞或骨髓中原始细胞≥5％（除外巩固化疗后骨髓再生等其他原因）或髓外出现白血病细胞浸润。难治性AML诊断标准：经过标准方案治疗2个疗程无效的初治病例；CR后经过巩固强化治疗，12个月内复发者；12个月后复发但经过常规化疗无效者；2次或多次复发者；髓外白血病持续存在者。纳入标准如下：①符合复发/难治AML（除外M_3_）诊断标准；②年龄<18岁；③预期生存期≥8周；④与监护人充分沟通并获得同意，同时签署知情同意书；8岁以上儿童告知本人，并签署知情同意书。全部入组患儿均采用CLAG-M/I方案治疗。本研究得到南京医科大学附属儿童医院医学伦理委员会的批准（批件号：202004019-1）。

2. 治疗方案：所有患儿均接受1～2个疗程CLAG-M/I方案化疗。考虑到阿糖胞苷与克拉屈滨联用有严重的骨髓抑制作用，我们对成人CLAG方案进行了调整，选择了复发难治性AML中国诊疗指南[2]推荐的最小阿糖胞苷剂量。CLAG-M/I方案具体包括：克拉屈滨5 mg/m^2^，静脉滴注（静滴）2 h，第1～5天；阿糖胞苷1 g/m^2^，静滴，第1～5天；G-CSF 5 µg/kg，皮下注射，第0～5天（以化疗前1天为第0天）；米托蒽醌或伊达比星8 mg/m^2^，静滴，第1～3天。研究后期由于米托蒽醌无法供应，我们用伊达比星代替米托蒽醌治疗部分患者。

1个疗程获得CR的患者，尽快给予异基因造血干细胞移植（allo-HSCT）；1个疗程未缓解（NR）或部分缓解（PR）的患者，进行第2个疗程化疗，CR后尽快进行allo-HSCT；仍未获得CR的患者，退出该方案，开展个体化治疗。

3. 疗效与安全性评估：CR：骨髓增生正常，幼稚细胞≤5％，流式细胞术（FCM）微小残留病（MRD）转阴；PR：骨髓增生正常，幼稚细胞6％～19％；NR：骨髓幼稚细胞≥20％。疗效评估在治疗的第21天或第28天进行。如第21天达CR，则不需要进行第28天评估。

安全性评估基于化疗的不良反应，根据WHO急性和亚急性毒副反应分度标准分为0～4度[4]。我们观察的不良反应包括骨髓抑制、感染、肝脏毒性以及胃肠道反应、皮肤黏膜损害等。

4. 随访：通过定期门诊就诊以及电话回访的方式进行随访。随访的内容包括治疗相关不良反应、疾病进展、复发或者死亡。末次随访时间为2021年9月22日。中位随访时间为16.8（2.3～27.9）个月。

## 结果

1. 患者的临床特征：9例患儿中，7例为难治性AML，2例为复发性AML（移植后）；男6例，女3例。中位发病年龄为8.25（1.7～13.5）岁。所有患儿既往均接受至少两个疗程常规化疗（蒽环类药物联合标准剂量阿糖胞苷）。MICM检查结果等临床资料见表1。

**表1:** 9例CLAG-M/I方案治疗复发/难治急性髓系白血病（AML）患儿的临床资料

例号	性别	初诊年龄（岁）	FAB分型	初诊WBC（×10^9^/L）	染色体核型	融合基因	AML相关突变
1	男	8.25	M_2_	103.80	46, XY[4]	阴性	WT、KRAS
2	男	11.9	M_4_	4.02	45, XY, −7[20]	阴性	CSF3R、KRAS、PTPN11、SETBP1、ASXL1、EVI1
3	女	5.0	M_5_	27.33	47, XX[13]	阴性	KRAS、EVI1
4	男	6.8	M_2_	81.49	46, XY, ? del（9）（q22）[6]/46, XY[14]	阴性	FLT3-ITD
5	男	12	M_4_	128.23	46, XY[15]	阴性	FLT3-ITD、WT1
6	女	13.5	M_1_	41.42	46-48, XX, ? t（2;17）（q31q25）, t（16;21）（p11; q22）[cp20]	TLS-ERG	BCORL1
7	男	1.6	M_1_	7.39	46, XY[20]	CBF2AT3-GLIS2	NOTCH1
8	女	9.9	M_2_	4.89	46, XX[20]	RUNX1-RUNX1T1	KIT
9	男	1.7	M_5_	23.68	47,XY,t（8:22）（p11.2:q13）,+der（8）t（8:22）[14]/46,XY[6]	阴性	KMT2CNM-170606.2

注：CLAG-M/I：克拉屈滨＋阿糖胞苷＋G-CSF＋米托蒽醌/伊达比星

2. 疗效：患儿的治疗反应如表2所示。使用CLAG-M/I方案1个疗程后，6例患儿CR；1例患儿PR，接受了第2个疗程治疗，并最终获得CR；7例CR患儿后续均进行了allo-HSCT。截至末次随访时间，7例患儿处于CR状态，例8复查骨髓细胞形态学及MRD均阴性，但出现RUNX1-RUNX1T1融合基因转阳，予以氟达拉滨及干扰素治疗过程中。

**表2:** CLAG-M/I方案用于儿童复发/难治急性髓系白血病的疗效

例号	诊断	治疗方案	疗效		allo-HSCT	转归^a^	存活时间（月）
			1个疗程	2个疗程			
1	难治	CLAG-M	CR	/	是	存活	28.5
2	难治	CLAG-M	PR	CR	是	存活	31.0
3	难治	CLAG-M	NR	/	是	死亡	10.6
4	难治	CLAG-I	CR	/	是	存活	20.4
5	难治	CLAG-I	CR	/	是	存活	18.8
6	复发	CLAG-I	CR	/	是	存活	27.8
7	难治	CLAG-I	NR	/	是	死亡	12.0
8	难治	CLAG-I	CR	/	是	存活	10.3
9	复发	CLAG-I	CR	/	是	存活	12.6

注：CLAG-M：克拉屈滨＋阿糖胞苷＋G-CSF＋米托蒽醌；CLAG-I：克拉屈滨＋阿糖胞苷＋G-CSF＋伊达比星；CR：完全缓解；PR：部分缓解；NR：未缓解；allo-HSCT：异基因造血干细胞移植；/：不适用。^a^截至2021年9月22日

2例患儿（例3和例7）在使用1个疗程CLAG-M/I方案后骨髓幼稚细胞比例持续增高，后续改为其他方案治疗仍未缓解，行脐血造血干细胞移植。例3最终由于疾病进展于确诊10个月后死亡；例7在移植后达到短暂缓解（114 d），但之后出现髓外联合骨髓复发，在诊断 AML 12个月后死亡。

3. 不良反应：我们分析了治疗过程中出现的所有不良反应，结果见表3。骨髓抑制是最常见的不良反应，表现为三系血细胞的减少。9例患儿均出现不同程度的骨髓抑制。3例患者表现为Ⅳ度贫血。Ⅳ度中性粒细胞和血小板减少的中位持续时间为22（18～44）d及26（18～44）d。

**表3:** 9例CLAG-M/I方案治疗复发/难治急性髓系白血病患儿的不良反应

例号	中性粒细胞恢复时间（d）	血小板恢复时间（d）	HGB下降	感染情况	抗生素使用时间（d）	肝脏损伤	胃肠道症状
1	20	18	Ⅳ度	龟头炎	9	Ⅰ度	Ⅲ度
2	20	26	Ⅳ度	脓毒血症	36	Ⅰ度	Ⅱ度
3	30	30	Ⅲ度	脓毒血症	25	Ⅱ度	无
4	18	19	Ⅲ度	脓毒血症	11	Ⅱ度	无
5	22	23	Ⅲ度	脓毒血症	12	无	无
6	30	31	Ⅲ度	脓毒血症	20	无	Ⅱ度
7	44	44	Ⅳ度	肛周脓肿	41	无	无
8	24	26	Ⅲ度	脓毒血症	23	无	Ⅰ度
9	22	22	Ⅲ度	脓毒血症	23	无	Ⅲ度

注：CLAG-M/I：克拉屈滨＋阿糖胞苷＋G-CSF＋米托蒽醌/伊达比星

9例患儿均出现粒细胞缺乏伴感染症状，其中例6出现感染性休克，给予积极抗休克治疗后很快好转。使用抗生素的中位时间为12（9～41）d。2例患儿（例1和例7）出现明显的皮肤黏膜损伤。例1化疗后出现阴茎红肿，有触痛，龟头分泌物中培养出白色假丝酵母菌，予制霉菌素涂抹、苯扎氯铵浸泡治疗后缓解。例7在化疗过程中出现肛周脓肿，予全身及局部抗感染治疗后好转。

胃肠道症状主要表现为呕吐、腹泻等症状，给予止吐、止泻等对症治疗后均好转。9例患儿转氨酶、胆红素等指标未见明显升高。

## 讨论

儿童AML患者异质性较大，预后往往较差，化疗耐药导致的复发和难治是其死亡的主要原因。尽管大多数指南建议此类患儿行allo-HSCT，但移植前骨髓达缓解是决定长期生存的关键[5]。

既往研究表明，克拉屈滨不仅具有直接的抗白血病作用，而且能够增加细胞内阿糖胞苷胞苷三磷酸（CTP）浓度，与阿糖胞苷协同抑制白血病细胞生长，最大程度地减少残留病[6]–[7]。2017年，Fridle等[8]采用CLA-Ida（克拉屈滨、阿糖胞苷、伊达比星）方案治疗成人复发AML患者，治疗相关死亡率为5.9％，CR率达52.9％。多项研究表明，CLAG-M/I方案在成人复发/难治AML中有显著的抗肿瘤活性和安全性，已被美国国立综合癌症网络（NCCN）指南列为成人复发/难治AML一线治疗方案[1],[8]–[10]。我国诊疗指南也建议对一般情况及耐受性好的复发/难治AML患者采用包括CLAG±M/I在内的强烈化疗方案[2]。

CLAG±M/I方案在儿童复发/难治AML中的临床使用经验相对较少。Ruan等[11]分别在20例和35例复发/难治AML患儿中比较了CLAG-M方案（克拉屈滨5 mg·m^−2^·d^−1^，阿糖胞苷2 g·m^−2^·d^−1^，G-CSF以及米托蒽醌）和 MEC/IEC方案（米托蒽醌/伊达比星、依托泊苷、阿糖胞苷）的疗效和不良反应，结果提示CLAG-M组（80％）的总体反应率（ORR）显著高于MEC/IEC组（51％）。Zhang等[12]采用改良CLAG（克拉屈滨9 mg·m^−2^·d^−1^，阿糖胞苷400 mg·m^−2^·d^−1^，G-CSF 5 µg·kg^−1^·d^−1^）方案治疗12例儿童复发/难治AML患者，1个及2个疗程后CR患儿分别为8例和9例；1例患儿死于严重感染。

考虑到化疗药物的不良反应，我们对成人CLAG方案[2]进行了减少剂量的调整：阿糖胞苷为1 g·m^−2^·d^−1^、米托蒽醌/伊达比星为8 mg·m^−2^·d^−1^。在使用1或2个疗程后，9例患儿中有7例获得CR，5例为难治性AML，2例为移植后复发AML。我们的缓解率与既往使用CLAG方案治疗儿童复发/难治AML的缓解率相近[11]–[12]，且未出现严重不良反应。最主要的不良反应为骨髓抑制和感染，但通过积极控制感染及支持治疗均恢复良好。

2例患儿使用CLAG-M/I方案后骨髓仍然未缓解，考虑到患儿存在原发耐药，可能与特殊的基因类型有关。例3 EVI1高表达，同时存在KRAS突变；例7存在CBF2AT3-GLIS2融合基因以及NOTCH1:NM-017617突变。已有研究报道上述基因异常极易导致原发耐药，预后极差。此类患者对多种化疗方案均不敏感，采用免疫治疗或靶向治疗，是今后研究的方向。Sadeghian等[13]揭示EVI1高表达与儿童AML预后无明显相关性；而KRAS基因和NOTCH基因过表达可降低AML患者的CR率以及总生存率[14]–[15]。对于原发耐药的患者，也可以通过人源肿瘤异种移植模型体内药效学检测（PDTX）等手段选择更为有效的化疗药物，为他们再次获得缓解提供机会。

对于儿童复发/难治AML，通过再诱导化疗获得CR后再行造血干细胞移植非常重要，而基于克拉屈滨的CLAG-M/I方案对于这些患儿来说是一个安全有效的化疗选择。

本研究的局限性在于样本量较小且随访时间较短。我们会对符合条件的复发/难治AML患儿继续采用CLAG-M/I方案诱导化疗，并对他们进行长期持续随访。
